# Diarrhea as a risk factor for acute lower respiratory tract infections among young children in low income settings

**DOI:** 10.7189/jogh.03.010402

**Published:** 2013-06

**Authors:** Christa L. Fischer Walker, Jamie Perin, Joanne Katz, James M. Tielsch, Robert E. Black

**Affiliations:** 1 Department of International Health, Johns Hopkins Bloomberg School of Public Health, Baltimore, MD, USA; 2 Department of Global Health, George Washington University, Washington DC, USA

## Abstract

**Background:**

Diarrhea and acute lower respiratory tract infections (ALRI) are leading causes of morbidity and mortality among children under 5 years of age. We sought to quantify the correlation of diarrhea and respiratory infections within an individual child and to determine if infection with one illness increases the risk of infection with the other during the same time period.

**Methods:**

We quantified the likelihood of an ALRI and a diarrhea episode occurring during the same week compared to the likelihood of each occurring independently in two cohorts of children under 3 years of age using a bivariate probit regression model. We also quantified the likelihood of an ALRI episode conditioned on a child’s diarrhea history and the likelihood of a diarrhea episode conditioned on a child’s ALRI history using Cox Proportional Hazard models.

**Results:**

In Indian and Nepali children, diarrhea and ALRI occurred simultaneously more than chance alone. Incidence of ALRI increased in both cohorts as the number of days with diarrhea in the prior 28 days increased; the greatest incident rate ratio was reported among children with 20 or more days of diarrhea (1.02, 95% confidence interval (CI) 1.01 – 1.03 in Nepal and 1.07, 95% CI 1.05 – 1.09 in South India). Incidence of diarrhea was affected differently by ALRI prevalence depending on season.

**Conclusions:**

Diarrhea may be a direct risk factor for ALRI among children under 3 years of age. The risk of comorbidity increases as disease severity increases, providing additional rationale for prompt community case–management of both diarrhea and pneumonia.

Pneumonia and diarrhea are the leading causes of death among children under 5 years of age around the world [1]. In low– and middle–income countries children under 5 years of age experience multiple episodes of diarrhea each year and 1.4 episodes of pneumonia before their fifth birthday [2]. These infectious diseases disproportionately cause severe morbidity and mortality among children living in high–risk populations typically characterized by poverty and inadequate health care.

There has long been speculation that some children may be more susceptible to simultaneous infections (ie, comorbidity) or may experience sequential infections because of compromised immune function and malnutrition. Although biologically plausible and clinically observable, there is little evidence quantifying the prevalence of common infectious disease comorbidities among children <3 years of age. Several studies have looked for the co–occurrence of diarrhea and pneumonia and have found that these two diseases occur together at a rate that is greater than that expected by chance [3-6].

We sought to quantify the correlation of diarrhea and respiratory infections within an individual child and determine if infection with one illness increases the risk of infection with the other during the same time period. By using cohort data, we are able to differentiate between overlapping infections and sequential infections and quantify the role of disease severity on each relationship.

## METHODS

### Selection of data sets

The analysis presented here was part of a wider portfolio of comorbidity analyses designed to quantify the risk relationships of overlapping infections, subsequent illnesses and mortality (not presented here). In this light we conducted a systematic literature review to identify studies with prospective morbidity and mortality surveillance among children under 5 years of age. We searched PubMed, Scopus, and Google Scholar for published studies between 1980 and 2010 using key words: diarrhea, pneumonia, acute lower respiratory tract infection (ALRI), morbidity and mortality. We excluded all studies that were not conducted among representative populations and had morbidity surveillance for less than 12 months. Included studies were required to have at least bi–weekly household morbidity surveillance to collect daily morbidity reports for all signs and symptoms of diarrhea and respiratory infections. We also contacted numerous investigators as part of the Child Health Epidemiology Reference Group (CHERG) (www.cherg.org) network of investigators by email and phone in an effort to identify ideal data sets.

We identified two studies that enrolled children under the age of 3 years for routine diarrhea surveillance in low– or middle–income countries. We identified one study from rural Sarlahi District in Southern Nepal (NNIPS–4 trial) that was designed as a cluster–randomized trial assessing the efficacy of preventive zinc and/or iron supplementation on morbidity and mortality [7]. All children from 1–23 months of age living in the study area were asked to participate in the main trial. Detailed morbidity data were ascertained during home visits weekly on a subset of infants from each treatment group for 12 months after enrollment.

We identified one study from South India that was designed as an individually randomized placebo-controlled trial (VASIN) assessing the efficacy of vitamin A supplementation for newborn infants [8]. Newborns were enrolled at birth and followed every two weeks until 6 months of age. Daily morbidity data were collected at each interview. For our analyses, we included only infants 1 month of age and older.

### Simultaneous correlation of ALRI and diarrhea episodes

We sought to quantify the likelihood of an ALRI and a diarrhea episode occurring during the same week compared to the likelihood of each occurring independently between two cohorts of children under 3 years of age. Daily morbidity records were summarized for each calendar week of morbidity surveillance, such that each week was classified as having no illnesses, a diarrhea episode, an ALRI episode, or both. Complete episode definitions are described in **Table 1**. Due to data limitations, we used the episode definitions as determined by the initial study investigators. We analyzed the weeks with both disease events occurring using a bivariate probit regression model. The bivariate probit analysis models both the individual prevalence of each outcome and the correlation between two simultaneous outcomes while assuming a latent normal variable [9]. The correlation within week is modeled explicitly with latent normality, while the correlation between observation weeks for the same child is accounted for in the standard errors with a robust covariance estimate. Comorbidity is quantified by the correlation of an episode of ALRI and diarrhea occurring during the same week. Given this, the correlation could range from –1, indicating an extreme inverse or protective relationship, to 1, which would indicate perfect co–occurrence of both illnesses. A value of zero would indicate that the co–occurrence of diarrhea and ALRI is by chance alone.

**Table 1:** Study definitions for diarrhea and respiratory morbidities

Disease	Nepal	South India
Diarrhea	≥4 loose watery stools/d, with episodes separated by ≥3 symptom–free days	≥4 loose watery stools/d, episodes separated by ≥3 symptom–free days
Persistent diarrhea	Diarrhea for ≥14 d	Diarrhea for ≥14 d
Dysentery	Blood or mucus in the stool, with ≥3 symptom–free days separating episodes	Blood or mucus in the stool, with >3 symptom–free days separating episodes
ALRI	Fever, cough, or difficulty breathing, with all 3 symptoms on ≥1 d during the episode with ≥7 d between episodes. Respiratory rates available from sub–study to define fast breathing: ≥60 breaths/min if <2 mo old; ≥50 breaths/min if 2–11 mo of age; and ≥40 breaths/min ≥12 mo	Below definitions of severity with episodes separated by ≥3 symptom–free days: ALRI–1: cough with fever on ≥1 d ALRI–2: difficulty breathing with fever on ≥1 d ALRI–3: cough and difficulty breathing with fever on ≥1 d

ALRI – acute lower respiratory tract infection, mo – month, d – day

For the NNIPS study data, we modeled the prevalence of ALRI and diarrhea by calendar month, age in four categories (1–5, 6–11, 12–23, and 24–35 months), sex and mid–upper arm circumference. For the VASIN data set, we controlled for calendar month and sex; we did not control for age given that age was between 1–5 months.

### Is recent morbidity predictive of ALRI or diarrhea incidence rates?

**Figure 1:**
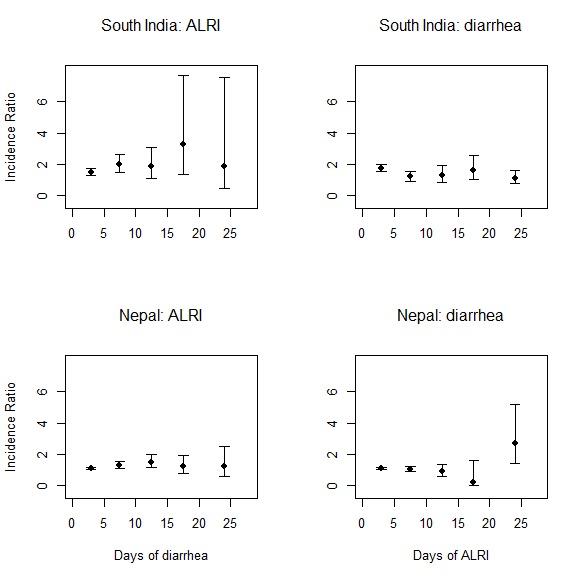
In the models for acute lower respiratory tract infection (ALRI) incidence, estimated incidence ratios for categorized diarrhea in the past 28 days, and in the models for diarrhea incidence, estimated incidence ratios for categorized history of ALRI in past 28 days.

We quantified the likelihood of an ALRI episode conditioned on a child’s diarrhea history using daily morbidity data from the two cohorts of children. We used daily morbidity data to define the presence of an ALRI episode for each day the child was enrolled in the study such that on each day a child was either positive or negative for ALRI. We then calculated the number of days the child had an episode of diarrhea in the 28 days prior to the index day. For any day with missing data we assumed the child did not experience diarrhea, unless there was no history of diarrhea available for any day in the last 28 days in which case the day was dropped from the analysis. We then used the Prentice–Williams–Peterson model that extends Cox proportional hazards regression to recurring events to estimate the incidence rate of ALRI conditional on the number of days of diarrhea in the previous 28 days [6,10,11]. We chose calendar time as the scale for this model in order to control for seasonal variation in the rates of respiratory infection, assuming the main effect of one additional day of diarrhea in the past four weeks proportional to the rate of ALRI, and that this effect would remain the same over time. In order to best summarize the relationship between recent diarrhea and incident ALRI, several functions of the number of recent diarrhea days were considered. We organized the number of days with each illness into categories and thus approximately linear relationships were observed in each study (**Figure 1**). In addition, the individual ALRI rate was used to group children for analysis, where incidence is estimated for each group and effects are estimated across groups. This stratification compares the number of illness days for children with a similar number of episodes. To address the potential for common risk factors of diarrhea and ALRI, we included age, sex and mid upper arm circumference (MUAC) where available for each child as covariates in this model. We initially included the proportion of time–spent ill for each child, but due to colinearity this violated basic model assumptions. For graphical purposes, we calculated an average monthly incidence rate, by calendar month for each additional day of diarrhea. To illustrate the risk relationship we plotted 4 risk relationships from the results of this model with a linear effect of recent diarrhea on ALRI incidence: 1) no diarrhea in previous 28 days; 2) 5 days of diarrhea; 3) 10 days of diarrhea and 4) 20 days of diarrhea.

Similarly, we estimated the likelihood of a diarrhea episode conditioned on a child’s history of ALRI. We used the same episode definitions for ALRI and diarrhea as described above. Using the daily data to define each index day as positive/negative for diarrhea, we then calculated the number of days of ALRI in the previous four weeks. For any day without a morbidity record, we assumed no ALRI (as also described above). We then modeled the incidence rate of diarrhea conditional on the number of days of ALRI in the last 28 days. We assumed that this ratio remains constant over time. As we described above we calculated the average monthly incidence rate by calendar month and plotted four risk relationships for different levels of recent ALRI rates as described above.

## RESULTS

### Simultaneous correlation of ALRI and diarrhea episodes

Among 4865 Nepali children, caregivers reported 6.7% of weeks with an episode of diarrhea and 3.2% of weeks with ALRI symptoms. In this population diarrhea and ALRI were also commonly reported as simultaneous comorbidities in 0.4% of weeks. Analysis with the bivariate probit regression model showed that diarrhea and ALRI have a small positive correlation and occurred together more than chance alone (0.15, 95% confidence interval (CI) 0.13 – 0.17) (**Table 2**). The effect was more pronounced among episodes of ALRI with elevated respiratory rates. Statistical adjustment with seasonality and child level covariates (age, sex, MUAC) did not change the positive correlation (**Table 2**).

**Table 2:** Correlation between weekly ALRI and diarrhea episodes as observed in the bivariate probit regression models among Nepali children and South Indian infants

	Estimate	Lower Limit	Upper Limit	Standard Error
**Nepali Children**				
Unadjusted:	0.153	0.132	0.174	0.011
ALRI & diarrhea				
ALRI & diarrhea with elevated respiratory rate	0.296	0.149	0.431	0.072
Adjusted:*				
ALRI & diarrhea	0.146	0.125	0.167	0.011
ALRI & diarrhea with elevated respiratory rate	0.250	0.092	0.395	0.078
**South Indian Infants**				
Unadjusted:				
ALRI–1& diarrhea	0.150	0.130	0.171	0.011
ALRI–2 & diarrhea	0.190	0.163	0.227	0.016
ALRI–3 & diarrhea	0.170	0.134	0.203	0.017
Adjusted:†				
ALRI–1& diarrhea	0.150	0.128	0.169	0.011
ALRI–2 & diarrhea	0.190	0.161	0.225	0.016
ALRI–3 & diarrhea	0.170	0.132	0.201	0.018

ALRI – acute lower respiratory tract infection, ALRI–1: cough with fever on ≥1 day, ALRI–2: difficulty breathing with fever on ≥1 day, ALRI–3: cough and difficulty breathing with fever on ≥1 day

*Adjusted for calendar time, age, sex, and mean upper arm circumference (MUAC).

†Adjusted for calendar time, sex.

Among 11 115 South Indian infants, caregivers reported 3.9% of weeks with an episode of diarrhea and 9.5% of weeks with ALRI symptoms classified as ALRI–1, 2.0% of weeks with ALRI symptoms classified as ALRI–2, and 1.6% of ALRI symptoms classified as ALRI–3. In this population of young infants, 0.6% of weeks were classified as having diarrhea and ALRI comorbidity symptoms. The bivariate probit regression model showed that diarrhea and ALRI symptoms (of any ALRI definition) occur together more than chance alone with the strongest association being between diarrhea and ALRI as defined by difficulty breathing and a fever (ALRI–2), with an estimated correlation of 0.19 (95% CI 0.16 – 0.23) (**Table 2**). There were no differences observed between the unadjusted correlation and the adjusted correlation (**Table 2**).

### Diarrhea and/or ALRI as a risk factor for subsequent morbidities

**Figure 2:**
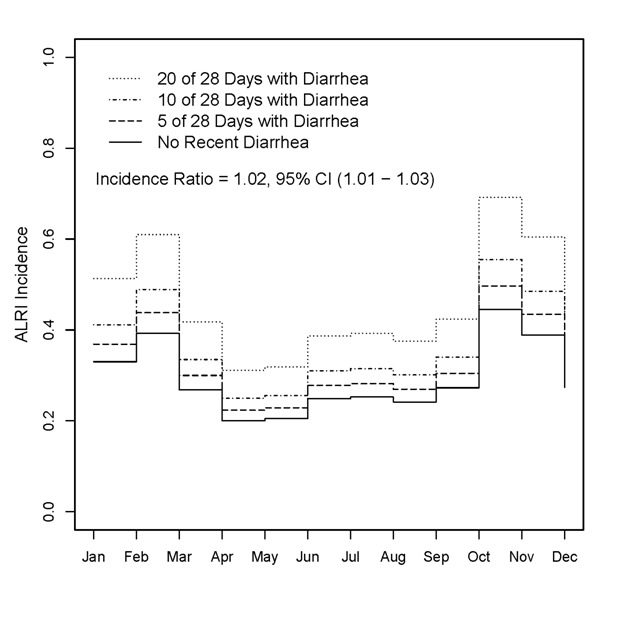
Estimated acute lower respiratory tract infection (ALRI) incidence by month and history of diarrhea in past 28 days for Nepali children.

We first calculated the incidence of ALRI by prior 28–day diarrhea history using the Prentice–Williams–Peterson Proportional Hazards model among Nepali children. These children reported an overall incidence rate of 1.4 episodes defined by reported signs or symptoms of ALRI per child year of observation. The predicted incidence rate increased as the number of days with diarrhea in the 28 days prior increased with the highest ALRI incidence rates predicted for children who reported 20 or more days of diarrhea in the preceding 28 days. The incidence ratio was modeled as a linear function of the number of days with diarrhea in the past 28, where the incidence ratio for one additional day of diarrhea was estimated at 1.02 (95% CI 1.01– 1.03) (**Figure 2**). This relationship was constant across seasons.

**Figure 3:**
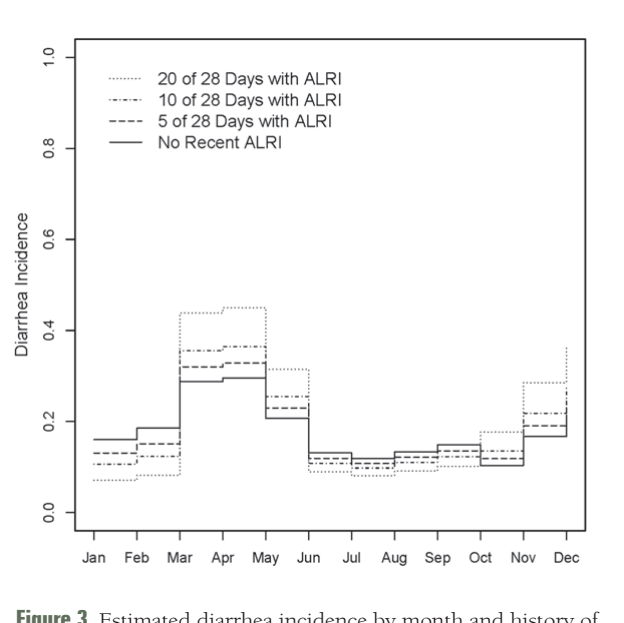
Estimated diarrhea incidence by month and history of acute lower respiratory tract infection (ALRI) in past 28 days for Nepali children.

We used the same methodology to assess the reverse relationship, ie, diarrhea incidence as a function of the signs and symptoms of ALRI in the preceding 28 days. Unlike the above analysis, the diarrhea incidence rate was affected differently by prevalence of ALRI symptoms depending on the time of year. The overall estimated linear effect of recent diarrhea days on ALRI incidence was negligible and not statistically significant, however, a Wald test for an interaction of effect with season was significant at alpha = 0.05. In the summer (March – May), diarrhea incidence rate was higher for those with ALRI symptoms (1.02, 95% CI 1.00 – 1.04), however, in other months, diarrhea incidence did not vary significantly by prevalence of prior ALRI symptoms (**Figure 3**).

**Figure 4:**
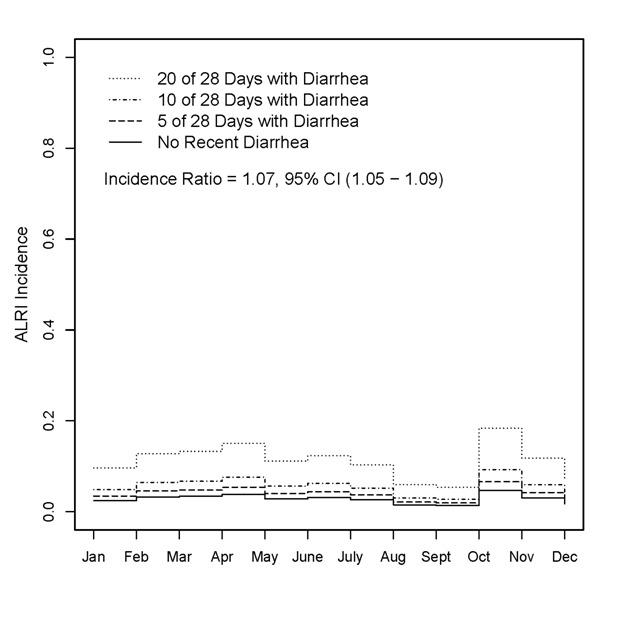
Estimated acute lower respiratory tract infection (ALRI) incidence by time and history of diarrhea in past 28 days among South Indian infants.

**Figure 5:**
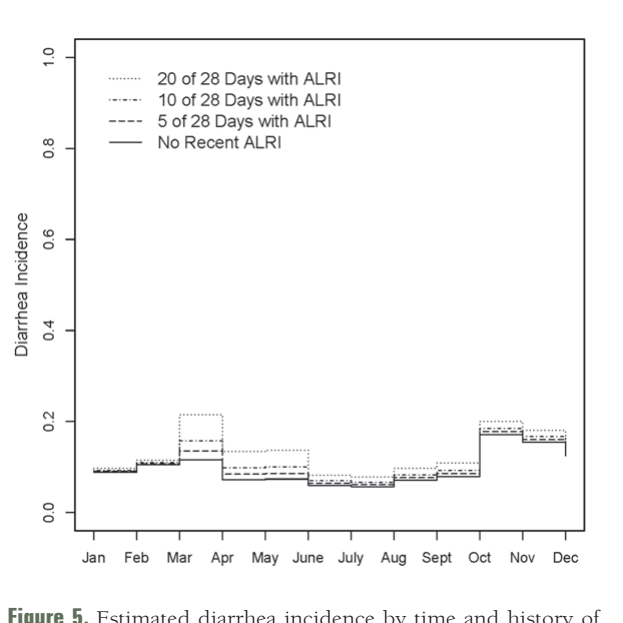
Estimated diarrhea incidence by time and history of acute lower respiratory tract infection (ALRI) in past 28 days among South Indian infants

We then calculated the incidence of ALRI (the most severe of the 3 ALRI definitions presented earlier) by 28–day diarrhea history among South Indian infants as was done in the Nepali children. Among these infants, the overall incidence rate of ALRI per child–year of observation was 0.3 episodes/child year. For these young babies an increase in the number of days with diarrhea was predictive of increased ALRI incidence with the highest ALRI incidence rates predicted for babies with 20 or more days of diarrhea in the past 28 days. For one additional day of diarrhea, the estimated incidence ratio was 1.07 (95% CI 1.05 – 1.09) (**Figure 4**). ALRI history was also more predictive of an increased rate of diarrhea with the highest diarrhea incidence rates predicted among infants with more than 20 days of ALRI in the preceding 28 days, but only in the summer (March–May, incidence ratio 1.03, 95% CI 1.01 – 1.06) and the monsoon season (June–September, incidence ratio 1.02, 95% CI 1.00 – 1.03) (**Figure 5**). Like the analysis using the Nepali data, the test for interaction between number of recent ALRI days and season was significant at alpha = 0.05, so the model with a constant effect of recent ALRI days on diarrhea incidence over time is not shown.

## DISCUSSION

We conducted two analyses to determine the relationship of ALRI and diarrhea among young children in Nepal and South India. In these low–income settings, we assessed the presence of diarrhea and ALRI alone as well as the likelihood of both occurring during the same week. In both populations, we found that ALRI and diarrhea present as simultaneous comorbidities more than would be expected by chance alone. This correlation increased in strength as severity of infection increased. In the second analysis we found that children in both countries who experienced more days of diarrhea had increased risk for subsequent ALRI episodes. However, the risk of diarrhea contingent upon prevalence of ALRI was only observed in some seasons.

Fenn et al [12] conducted a similar analysis using the bivariate probit regression model to quantify the simultaneous comorbidity among a cohort of children in rural Ghana. They too observed simultaneous comorbidity with diarrhea and ALRI occurring more than chance alone, and were also able to quantify this for varying degrees of disease severity. The correlation of combined infection increased as disease severity increased. In the Nepal and South Indian data sets, we were not able to differentiate episodes into disease severity beyond the 3 categories presented in the South Indian cohort. Future comorbidity analyses will benefit from detailed morbidity data such that correlations between multiple categories of disease severity can be assessed.

We conducted our analysis utilizing two large cohort studies that enabled us to assess the longitudinal daily prevalence of both diarrhea and ALRI. This permitted us to analyze ALRI as a risk factor for diarrhea and vice versa. Schmidt et al [6] conducted a similar analysis among Ghanaian and Brazilian children and found diarrhea prevalence to be a risk factor for ALRI among Ghanaian children, but not among Brazilian children. They postulated that this difference could be attributed to large differences in socioeconomic and epidemiologic conditions between the two populations; as could also be observed by the 10–fold difference in mortality rates between the two study populations. Coles et al [13] also observed diarrhea to be a risk factor for community–acquired alveolar pneumonia in Bedouin children in Israel. In our analysis of Nepali and South Indian children, we found the risk relationship to be much stronger for diarrhea as a risk factor for ALRI as compared to ALRI as a risk factor for diarrhea, which was significant only in certain seasons or among the South Indian infants.

There are several possible mechanisms or possible explanations for simultaneous and sequential infections. While it is possible that the signs and symptoms of diarrhea can mimic those of pneumonia, eg, severe diarrhea and dehydration can lead to metabolic acidosis and result in rapid, shallow breathing, it is an unlikely explanation for our findings [14]. First, diarrhea of that severity was rare in the studies analyzed and second, this would not explain the relationship with the respiratory illness following diarrhea, rather than being simultaneous with it; simultaneous infections were only recorded in less than 1% of observed weeks. It is well known that diarrhea can often lead to nutritional setbacks and hence the start of a cycle of illness, growth failure, and subsequent developmental delays [15]. These analyses suggest that the same cycle may not be observed given an episode of ALRI and thus the longer–term effects on growth, development, and immune function may differ between diarrhea and ALRI. One possible explanation for this effect is the loss of zinc during diarrhea, increasing the occurrence of zinc deficiency which in turn increases the risk of both ALRI and diarrhea [16]. Beyond the biological explanations for comorbidity, it is also possible that children with one infection are at greater risk for subsequent infections introduced at the point of care–seeking by increasing contact with children carrying other pathogens. It has also been observed that children living in the poorest houses are more exposed to infection as a result of lack of access to prevention and treatment interventions and thus sick children tend to live and play around other sick children exacerbating the cycle of infection [17].

Our analyses had a number of strengths. The study populations are both representative of rural low–income settings in South Asia, but provide two unique perspectives with regard to possible variation of comorbidity by age. The weekly prevalence of disease differed between the two populations, as did the relationship between diarrhea and ALRI comorbidity. This might be explained simply by anticipating variation when conducting analyses between two unique populations or might be more strongly related to the differences in age. However, our goal was not to compare one population to the other, rather to look for consistencies in the directionality and magnitude of the effects. Because these cohort studies used active community–based survey teams to gather morbidity data, we are able to look at the natural history of disease and determine how each individual illness affects the child’s health in the subsequent weeks and months.

Our analyses also have a number of limitations. As with any secondary analysis, we were limited to the data that had already been collected several years ago and thus not able to perform sub–group analyses by disease severity, or to control for common risk factors of ALRI and diarrhea beyond what was originally collected. Specifically, counted respiratory rates as well as X–ray confirmation of pneumonia for all children would have improved the case definition of ALRI and led to better associations of the relationship between diarrhea and pneumonia, in lieu of more general ALRI episodes. In addition, because these studies primarily gathered all information on the child’s signs and symptoms using household interviews with 1–2 week recall periods, the sensitivity and specificity of defining an episode has limitations. A caregiver might forget to recall minor episodes from several days prior, an episode could escalate in the days immediately following the interview and not be captured well during the next interview, or caregivers may forget several signs and symptoms over time. While daily surveillance of signs and symptoms would permit such measurements as counted respiratory rates and temperature, these frequent visits can also alter the natural history of disease by increasing the likelihood of care–seeking through early case detection and active referral reducing the ability to find a relationship. In addition, differences in recall period (7–day recall in Nepal vs 14–day recall in South Asia) may have also introduced bias.

Where daily data were missing, we assumed no disease for either diarrhea or ALRI. This may be underestimating incidence and days ill for both diseases, but given higher incidence rates of diarrhea, this would be more pronounced for diarrhea. Though this is a limitation, the effect would be an underestimation of the relationship between diarrhea and ALRI.

Lastly, in our analysis we only considered the short–term associations between diarrhea and ALRI. Further analyses of the longer–term associations that should include detailed anthropometric data in addition to disease surveillance are needed. We still do not fully understand the relationship of multiple infections either occurring together or in sequence during a very short time period, the results of these analyses suggest that comorbidity is a quantifiable problem and should be further investigated to better understand the full consequences of disease for both diarrhea and ALRI.

In the analysis here and in past reports [6,13], diarrhea has been shown to be an important risk factor for ALRI and it might be hypothesized that it may also be an important risk factor for ALRI/pneumonia cause–specific mortality, but further studies are needed to make the leap from morbidity to mortality. When calculating burden of disease, episodes and deaths from diarrhea and pneumonia are typically not considered as risk factors or contributing causes which may underestimate the true disease burden of these infectious diseases. This is becoming especially apparent for diarrhea for which the cycle of infection and undernutrition has long been recognized, but not fully quantified [15]. These new analyses further suggest the role of diarrhea as a direct risk factor for ALRI and highlight the importance of additional research to more fully understand the immunologic and biochemical mechanisms of this relationship.
